# The burden of illness in patients with paroxysmal nocturnal hemoglobinuria receiving treatment with the C5-inhibitors eculizumab or ravulizumab: results from a US patient survey

**DOI:** 10.1007/s00277-021-04715-5

**Published:** 2022-01-01

**Authors:** David Dingli, Joana E. Matos, Kerri Lehrhaupt, Sangeeta Krishnan, Michael Yeh, Jesse Fishman, Sujata P. Sarda, Scott B. Baver

**Affiliations:** 1 Mayo Clinic, Rochester, MN USA; 2 Kantar Health, New York, NY USA; 3 Apellis Pharmaceuticals, Inc., Waltham, MA USA

**Keywords:** Paroxysmal nocturnal hemoglobinuria, Eculizumab, Ravulizumab, Fatigue, Quality of life, Burden of illness

## Abstract

Paroxysmal nocturnal hemoglobinuria (PNH) is a rare and life-threatening disease with symptoms of hemolysis and thrombosis. Current therapies for this complement-mediated disease rely predominantly on inhibition of the C5 complement protein. However, data on treatment responses and quality of life in C5-inhibitor (C5i)-treated PNH patients are scarce. The objective of this study was to determine C5i treatment effects on clinical parameters, PNH symptoms, quality of life, and resource use for PNH patients. This cross-sectional study surveyed 122 individuals in the USA receiving treatment for PNH with C5-targeted monoclonal antibodies, eculizumab (ECU) or ravulizumab (RAV). Despite most patients receiving C5i therapy for ≥ 3 months (ECU 100%, *n* = 35; RAV 95.4%, *n* = 83), many patients remained anemic with hemoglobin levels ≤ 12 g/dL in 87.5% (*n* = 28/32) and 82.9% (*n* = 68/82) of ECU and RAV recipients, respectively. A majority of patients on ECU (88.6%; *n* = 31/35) and RAV (74.7%; *n* = 65/87) reported fatigue symptoms. Among PNH patients receiving C5i therapy for ≥ 12 months, some still reported thrombotic events (ECU, 10.0%, *n* = 1/10; RAV, 23.5%, *n* = 4/17) and required transfusions within the past year (ECU, 52.2%, *n* = 12/23; RAV, 22.6%, *n* = 7/31). Other patient-reported PNH symptoms included breakthrough hemolysis, shortness of breath, and headaches. Patients reported scores below the average population norms on the Functional Assessment of Chronic Illness Therapy (FACIT)-Fatigue and European Organization for Research and Treatment of Cancer Quality of Life Questionnaire Core 30 (EORTC QLQ-C30) scales. Overall, this study found that PNH patients receiving ECU or RAV therapy demonstrated a significant burden of illness, highlighting the need for improved PNH therapies.

**Supplementary Information:**

The online version contains supplementary material available at 10.1007/s00277-021-04715-5.

## Introduction

Paroxysmal nocturnal hemoglobinuria (PNH) is a rare, acquired, and life-threatening disease characterized by the chronic lysis of red blood cells (RBCs) and a high propensity for thrombosis [1]. PNH is caused by a somatic mutation in the phosphatidylinositol glycan class A gene (*PIG-A*) in hematopoietic cells [2, 3]. This gene encodes for a protein that enables cells to synthesize glycosylphosphatidylinositol (GPI) anchors, which are covalently linked protein modifications to anchor proteins on the cell surface [2, 3]. Cells lacking GPI-anchored complement regulatory proteins, such as CD55 and CD59, are subject to destruction and lysis by the complement system.

The somatic mutation associated with PNH leads to complement-mediated hemolysis in patients with PNH, usually resulting in anemia [4]. The hemolysis that occurs in this disease has two distinct mechanisms: intravascular hemolysis (IVH) and extravascular hemolysis (EVH). IVH occurs when RBCs are directly lysed due to the activation of the alternative complement pathway [5]. This involves the formation of complexed complement proteins such as C3 convertase, C5 convertase, and the membrane attack complex (MAC). EVH occurs when RBCs are opsonized by fragments of the complement protein C3, which targets RBCs for phagocytosis in the spleen and liver [6].

Individuals living with PNH experience clinical manifestations of the disease such as anemia, thrombosis, hemoglobinuria, renal insufficiency, and bone marrow failure [1, 7]. Other common symptoms include fatigue, dyspnea, abdominal pain, and chest pain [7]. These symptoms can negatively impact the quality of life (QoL) of patients with PNH. One 2019 study found severe levels of fatigue and decreased QoL in individuals living with PNH [8]. Additionally, patients with PNH who were dependent on transfusions showed the lowest QoL scores [8]. Multivariable analysis results from this study also demonstrated that hemoglobin (Hb) levels and comorbidities had a major impact on QoL scores in patients living with PNH [8].

Current complement treatment options for PNH rely predominantly on C5-inhibition (C5i) with the monoclonal antibodies eculizumab (ECU) or ravulizumab (RAV). Both ECU and RAV bind to C5 and inhibit the activation of the terminal complement cascade, thus preventing MAC formation and IVH. RAV differs from ECU via modifications that extend its half-life [9]. Patients with PNH who are treated with ECU or RAV show improvements in disease symptoms [10–12]. However, disease manifestations that are related to EVH and mediated by the C3 complement protein may occur in patients receiving C5i therapy; in fact, one study reported that 72% of patients on C5-targeted ECU therapy remained anemic, with 36% of PNH patients receiving ECU requiring one or more transfusion per year [13]. Furthermore, C5i may also contribute to the low-level EVH observed in some ECU-treated patients, as it has been demonstrated that the blockade of C5 may unmask EVH by allowing for the accumulation of C3 fragments on PNH RBCs [14].

Although some findings suggest that patients with PNH who are treated with C5i still experience symptoms of the disease [13], the patient experience and reported QoL among patients with PNH treated with more recently available therapies have not been well documented in a real-world setting. Furthermore, data on clinical responses to treatment using validated QoL instruments in patients with PNH who are treated with C5i are scarce. Evidence suggests that data gathered from patient-reported outcome measures, including validated QoL scales, have the potential to inform patient care and benefit outcomes for patients with rare diseases [15, 16]. Given these observations, the objectives and research question for the current study aimed to evaluate and report the medical care and living experience of C5i-treated patients with PNH, as measured by self-reported clinically relevant laboratory parameters and PNH symptoms, resource use questions, and validated QoL instruments. Accordingly, a cross-sectional patient survey was developed to measure the clinical, humanistic, and economic burden of illness among patients in the USA with a self-reported diagnosis of PNH and receiving therapy with ECU or RAV.

## Methods

### Survey development

A cross-sectional survey was developed using a set of criteria to select the measures that were utilized to query patients with PNH. Each measure was (1) designed to address the relevant clinical and humanistic domains of interest in PNH, (2) formulated to allow for standardized collection, and (3) based on information that would likely be known or available to the patient. The electronic survey used for data capture was a Health Insurance Portability and Accountability Act (HIPAA) compliant secure portal with built-in logic to ensure accuracy of patient responses. Before beginning the survey, patients were screened based on the following factors: survey taker, current treatment, and age. This was a cross-sectional survey that evaluated a single timepoint for each participant. Therefore, the survey was not repeatedly applied, and the values reported from this study represent dosing information, clinical parameters, PNH symptoms, and QoL assessments that were captured during a single timepoint within a given participant’s experience with PNH and C5i treatment.

### Ethical considerations

This study was conducted in accordance with the International Society for Pharmacoepidemiology (ISPE) Guidelines for Good Pharmacoepidemiology Practices (GPP) [17]. The General Data Protection and Regulation (GDPR) requirements were followed to protect patient data and privacy [18]. A study exemption from the US Central Institutional Review Board (IRB) was obtained before initiation of the survey. This study was in a category of exempt human studies research as it utilized survey procedures to gather data. In accordance with the Declaration of Helsinki ethical principles for medical research and the US Office of Human Research Protections (OHRP) guidance about informed consent, all participants electronically provided documentation of their informed consent as evidence of their agreement to voluntarily participate in the study. Additionally, participants agreed to the study sponsor’s adverse event reporting requirements [19, 20]. To protect patient privacy, the working data files used for analysis contained no specific patient identifying information, only an assigned panel ID number.

### Participants, survey study design, and recruitment procedures

Individuals aged 18 or older with a self-reported diagnosis of PNH who were receiving treatment for PNH with either ECU or RAV were recruited to participate in an online one-time, cross-sectional survey conducted between July and October 2020. The study was designed to be cross-sectional with the objective to evaluate the relationship between exposure to C5i and clinical parameters, PNH symptoms, and QoL-based outcomes. The sampling approach for this study combined purposive (i.e., a specific population) and convenience sampling in which individuals were recruited through the patient advocacy group Aplastic Anemia & Myelodysplastic Syndrome International Foundation (AAMDS). This foundation was considered to serve as an appropriate organization for the recruitment of patients with PNH because it is thought to represent a large portion of the PNH population in the USA. Email invitations with a unique link were sent to potential participants to take part in a one-time survey. Participants could use the unique link to enter and exit the survey as they chose.

Based on the sample size calculation and prevalence estimates of this rare condition [21], a minimum of 120 respondents were sought. The observation that survey response rates in rare disease populations are generally high [22] was also taken into consideration. The current sample sizes for the subgroups (ECU, *n* = 35; RAV, *n* = 87) were determined to provide at least 80% power and a significance level at *α* = 0.05 to detect an effect size Cohen’s *d* = 0.6. Following the informed consent process, participants were directed to the online cross-sectional survey instrument to complete the survey, and their responses were recorded into the secure research database. Any individuals reporting comorbidities such as multiple myeloma, hemophilia, leukemia, or lymphoma or who were unwilling to voluntarily agree to participate and record their informed consent were excluded from the study.

### Management of data and survey responses

Survey responses were collected into a HIPAA-compliant secure database that allowed for the direct exportation of the data into analytical software. Logic programming was incorporated into the survey prior to initiating the study to assure the integrity of the response dataset. These programming procedures included response ranges, consistency checks, skip patterns, and other special edit procedures where applicable. A response to all questions was required prior to a participant submitting the survey as completed, which ensured there was no missing data from respondents. Non-response notifications were built into the secure platform to alert the participant to complete any outstanding questions. Survey responses were reviewed and evaluated for several housekeeping criteria to ensure response and survey content validity. Criteria that flagged survey responses for scrutiny and individual evaluation included (1) patterned responses that were provided consistently throughout the survey, (2) identification of survey participants with a lack of credibility, and (3) participants that completed the survey in less than 10 min.

### Survey content

In total, 149 questions were used in the survey which were divided into 4 sections to collect information on parameters such as demographics (age, sex, weight), disease information (number of years with PNH, age at diagnosis), and treatment data (time since treatment initiation, dosage information), QoL using validated scales, and HCRU. Comorbidities were also assessed, which included aplastic anemia (AA) or severe aplastic anemia (SAA), myelodysplastic syndrome (MDS), and other bone marrow disorders. The survey was estimated to take approximately 25 min for a patient to complete and included questions regarding (1) patient-reported clinical parameters including most recent Hb levels, blood transfusion history, and thrombotic events; (2) current PNH symptoms including fatigue, breakthrough hemolysis, shortness of breath, headaches, difficulty focusing, and sleeping difficulties; (3) current QoL and fatigue using the psychometrically tested and validated European Organization for Research and Treatment of Cancer Quality of Life Questionnaire Core 30 (EORTC QLQ-C30) and Functional Assessment of Chronic Illness Therapy (FACIT)-Fatigue scales, respectively; (4) HCRU including number of PNH-related emergency room (ER) visits and hospitalizations in the last 12 months and PNH symptoms associated with HCRU; and (5) the Work Productivity and Activity Impairment (WPAI) Specific Health Questionnaire.

Clinical parameters (Hb levels, blood transfusion history, thrombotic events) were not confirmed by a physician. Participants were asked to select a multiple-choice answer (yes, no, not sure) to indicate whether they had ever experienced a blood transfusion or thrombotic event (blood clot). Individuals that answered “yes” were then asked to specify the frequency of these events in the past 12 months. To exclude blood transfusions and thrombotic events that may have occurred in the absence of complement-inhibitor therapy, patients that had not been on C5i therapy for ≥ 12 months were excluded from the blood transfusion and thrombotic event analyses. Similar to the collection of clinical parameters, current PNH symptoms reported by the study participants were not confirmed by a physician. Specifically, patients who reported breakthrough hemolysis as a current symptom of PNH were not asked to validate the occurrence of this symptom using laboratory values (i.e., lactate dehydrogenase levels).

General population values for the FACIT-Fatigue and EORTC QLQ-C30 assessments were derived from the available literature for comparison [23, 24]. The FACIT-Fatigue questionnaire is a 13-item assessment that collects information about the intensity of fatigue and how it impacts daily life [24]. The FACIT-Fatigue scale range is 0 (worst) to 52 (no fatigue), with 52 as the best possible score [25]. The EORTC QLQ-C30 assessment contains 30 items with five scales that assess functioning (physical, role, emotional, cognitive, and social) and one global health/QoL scale [23]. The EORTC QLQ-C30 score ranges between 0 and 100 with higher functional and global health scale scores indicating a better or healthier level of functioning [26]. Importantly, the FACIT-Fatigue and EORTC QLQ-C30 instruments have been validated for use in patients with PNH by a previous study [27]. The WPAI is a 6-question psychometrically validated instrument designed to be modified to a health problem by specifying the disease/condition in the questions [28]. Four scores are calculated from the WPAI: (1) absenteeism (percentage of work time missed in the past 7 days because of one's health problem [29]), (2) presenteeism (percentage of impairment experienced at work in the past 7 days because of one’s health problem [29]), (3) an overall work impairment score that combines absenteeism and presenteeism, and (4) the impairment in activities performed outside of work. Greater scores indicate greater impairment. Questions related to absenteeism and presenteeism were only asked among the employed survey participants. All values presented in this report for QoL, FACIT-Fatigue, HCRU, and WPAI represent the status and experience of the participants at the time of the survey. Individuals participating in this study were not asked to provide retrospective baseline values for these assessments that would represent their experience prior to C5i therapy.

This survey was made available to invited individuals in July 2020, approximately 4 months after the World Health Organization declared the outbreak of the novel coronavirus disease-2019 (COVID-19) a global pandemic. The impact of the COVID-19 pandemic was considered during the creation of this survey. For questions related to parameters that could be affected by the pandemic (treatment schedule including days between infusions and employment status), participants were asked to provide answers that described their experience prior to the dissemination of COVID-19.

### Analysis

Descriptive statistics were used to analyze the results from the survey. This included the calculation of means and standard deviations for continuous variables. Categorical variables were analyzed using frequencies and counts. According to the study protocol, the survey results were planned for and analyzed by stratifying the data corresponding to treatment type (ECU/RAV). ANOVA analyses were performed on continuous variables to identify significant differences between the two treatment subgroups, while chi-squared tests were conducted on categorical variables, where applicable. Statistical analyses were conducted using R version 3.5.3.

## Results

### Cohort demographics and dosage/treatment characteristics

Survey responses were recorded from 122 individuals with a self-reported diagnosis of PNH and who met the inclusion criteria for this study. The screening portion of the survey was initiated by 480 individuals, of which 298 (62.1%) quit during screening, and 66 (13.8%) were screened out based on failure to meet the inclusion criteria. Out of the remaining 126 individuals, 122 (96.8%) individuals met all the screening criteria and completed the survey. The demographics and PNH-specific disease characteristics for the survey participants are summarized in Table 1. Among the 122 participants that completed the survey, 35 (28.7%) were receiving ECU therapy and 87 (71.3%) were on RAV. The mean age (± SD) of the survey participants was 46.8 (± 15.7) years. The patients surveyed were 73.0% female (*n* = 89/122). On average, patients had been diagnosed with PNH for 9.2 (± 8.6) years at the time of participation in this study. The average weight of the cohort was 77.6 (± 19.5) kg. Aplastic anemia (AA)/severe aplastic anemia (SAA) affected 33.6% (*n* = 41/122) of the survey participants. Other comorbidities, such as myelodysplastic syndrome (MDS) or other bone marrow disorders, were reported by less than 6% (*n* = 7/122) of survey responders.

**Table 1:** Cohort demographics and dosage/treatment patterns

**Characteristic**	**Overall** (*N*^a^ = 122) **Mean (SD)**	**Eculizumab** (*n* = 35) **Mean (SD)**	**Ravulizumab** (*n* = 87) **Mean (SD)**
Age (years)	46.8 (15.7)	42.7 (15.9)	48.4 (15.4)
Age at diagnosis (years)	37.5 (14.5)	33.7 (15.7)	39.1 (13.8)
Number of years with PNH	9.2 (8.6)	9.0 (10.2)	9.3 (8.0)
Weight (kg)	77.6 (19.5)	78.1 (19.4)	76.5 (20.0)
Days between infusions (dosing frequency)	NA	17.1 (14.7)	55.0 (3.3)
	**n (%)**	**n (%)**	**n (%)**
Gender: Female	89 (73.0%)	28 (80.0%)	61 (70.1%)
Aplastic anemia/severe aplastic anemia	41 (33.6%)	10 (28.6%)	31 (35.6%)
Myelodysplastic syndrome	5 (4.1%)	3 (8.6%)	2 (2.3%)
Other bone marrow disorder	2 (1.6%)	1 (2.9%)	1 (1.1%)
Time since treatment initiation			
3 or more months 1 or more years	118 (96.7%) 89 (73.0%)	35 (100.0%) 31 (88.6%)	83 (95.4%) 58 (66.7%)
Frequency of patients taking higher than label recommended doses^b^		11 (31.4%)	2/6 (33.3%)^c^ 6/33 (18.2%)^d^

^a^*N* includes 1 respondent who chose not to identify for gender

^b^Higher than doses specified in US Food and Drug Administration package insert [30, 31﻿]

^c^Based on the number of patients in the 40–60 kg weight range

^d^Based on the number of patients in the 60–100 kg weight range

*Abbreviations*: *PNH*, paroxysmal nocturnal hemoglobinuria; *SD*, standard deviation

Survey participants with a self-reported diagnosis of PNH provided details of their dosage and treatment characteristics (Table 1). Therapy dosing frequency, measured in days between infusions, reflected the improved half-life of RAV compared to ECU with an average of 17.1 (± 14.7) days for ECU and 55.0 (± 3.3) days for RAV [9]. The majority (96.7%, *n* = 118/122) of patients had been treated with RAV or ECU for 3 or more months at the time of the survey. Furthermore, 88.6% (*n* = 31/35) of the ECU users and 66.7% (*n* = 58/87) of the RAV users reported that they had received therapy for 1 or more years. When asked about their therapeutic dosage, 31.4% (*n* = 11/35) of ECU users reported receiving higher than the US label recommended dose (> 900 mg) [30]. For RAV users, 33.3% (*n* = 2/6) of the patients in the 40–60 kg body weight category, and 18.2% (*n* = 6/33) of the patents in the 60–100 kg range reported higher doses than the US label recommended dose of RAV for their weight (40–60 kg: > 3000 mg, 60–100 kg: > 3300 mg) [31].

### Patient-reported clinical parameters and current symptoms

**Fig. 1:**
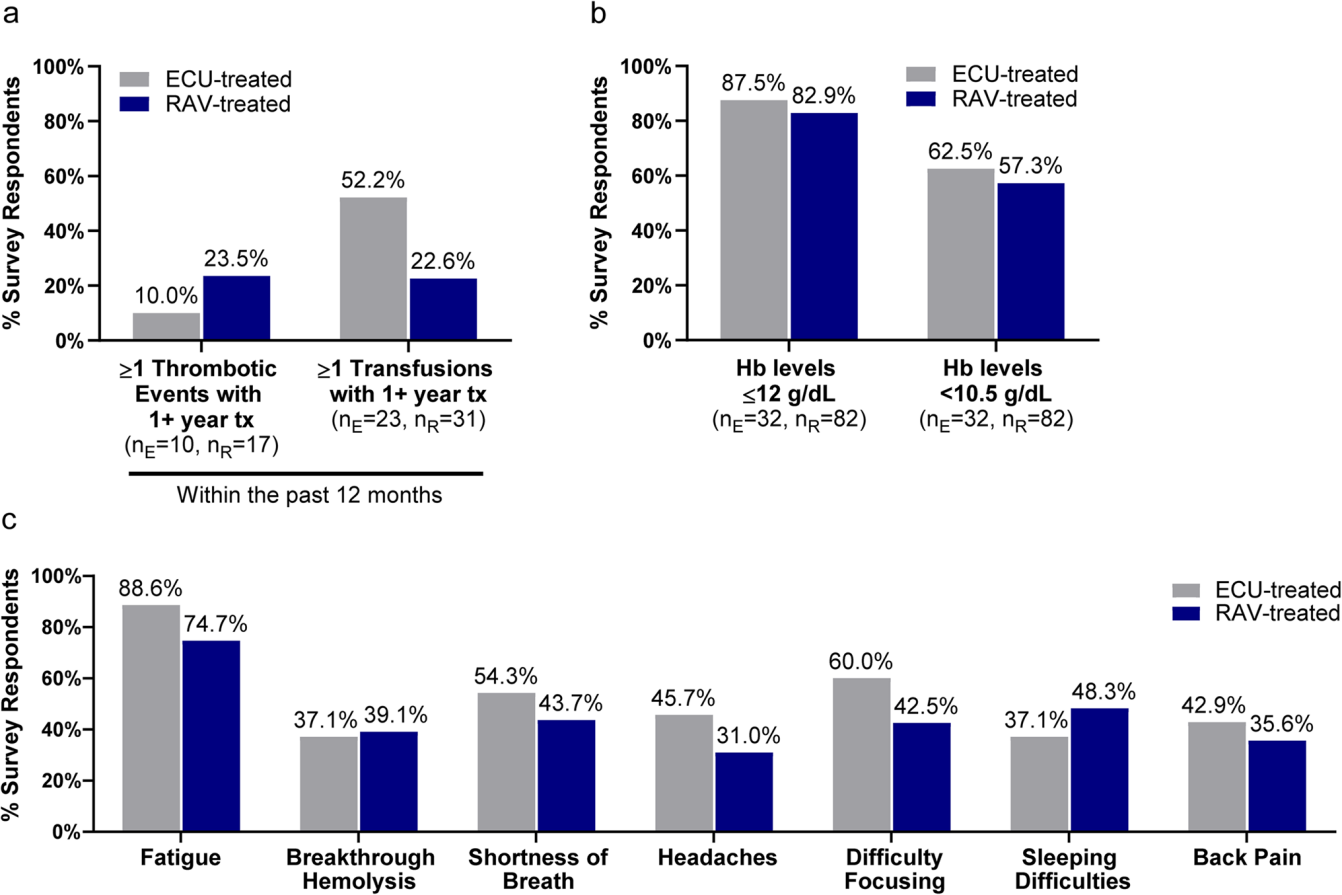
Patient-reported clinical parameters and PNH symptoms. **a** Number of thrombotic events and transfusions reported by survey participants within the past 12 months. *N* reported here represents the number of individuals that had at least experienced a thrombotic event/transfusion once in their lifetime and who were on C5i treatment (ECU or RAV) for one or more years. **b** Most recent patient-reported Hb levels. *N* represents the number of survey participants that reported their most recent Hb levels (overall, *n* = 114). **c** Most frequently reported current PNH symptoms that were disclosed by ≥ 35% of total survey participants (*N* = 122). *Abbreviations*: *C5i*, C5-inhibitor; *ECU*, eculizumab; *Hb*, hemoglobin; *n*_*E*_, *n* for ECU users; *n*_*R*_, *n* for RAV users; *PNH*, paroxysmal nocturnal hemoglobinuria; *RAV*, ravulizumab; *Tx*, treatment

Survey respondents who had received one or more years of treatment with either C5i, and who had experienced at least one thrombotic event/one transfusion in their lifetime, were asked about their history of thrombotic events and transfusions in the past 12 months. Thrombotic events were reported by 10.0% (*n* = 1/10) of ECU users and 23.5% (*n* = 4/17) of RAV users (Fig. 1a). The need for one or more transfusions was disclosed by 52.2% (*n* = 12/23) of individuals receiving ECU therapy and 22.6% (*n* = 7/31) of individuals receiving therapy with RAV (Fig. 1a).

The survey showed that among participants who provided Hb levels (*n* = 114), most C5i-treated individuals reported Hb levels ≤ 12 g/dL (ECU, 87.5%, *n* = 28/32; RAV, 82.9%, *n* = 68/82) (Fig. 1b). More than half of the survey participants who provided Hb levels reported levels < 10.5 g/dL despite treatment with ECU or RAV (ECU, 62.5%, *n* = 20/32; RAV, 57.3%, *n* = 47/82) (Fig. 1b).

Figure 1c shows the most common and current PNH symptoms reported by at least 35% of the total survey respondents. These PNH symptoms include fatigue, breakthrough hemolysis, shortness of breath, headaches, difficulty focusing, sleeping difficulties, and back pain. The most common symptom reported was fatigue (ECU, 88.6%, *n* = 31/35; RAV, 74.7%, *n* = 65/87) (Fig. 1c). Further analysis demonstrated that individuals with Hb levels < 10.5 g/dL reported significantly higher frequency of fatigue (86.6%, *n* = 58/67, *p* = 0.004) and breakthrough hemolysis (52.2%, *n* = 35/67, *p* < 0.001) as compared to patients with Hb levels ≥ 10.5 g/dL (fatigue: 63.8%, *n* = 30/47; breakthrough hemolysis: 19.1%, *n* = 9/47).

### Quality of life analysis

**Fig. 2:**
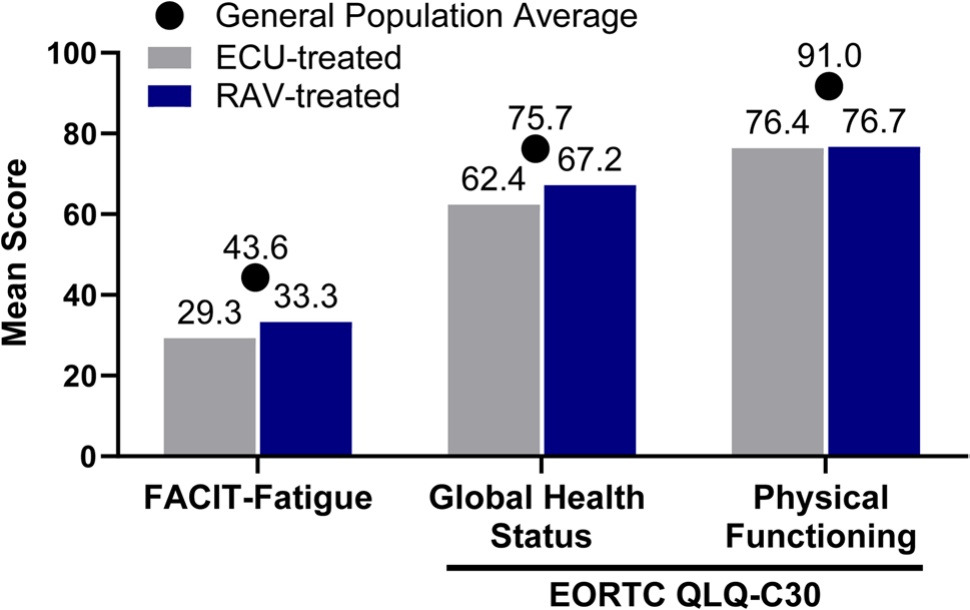
FACIT-Fatigue and EORTC QLQ-C30 scores. Mean FACIT-Fatigue score gathered from patients with PNH receiving C5i therapy (*N* = 122) compared to FACIT-Fatigue score for the general US population [24]. Mean EORTC-QLQ-C30 scores for global health status and physical functioning recorded from C5i-treated patients with PNH (*N* = 122) compared to the EORTC QLQ-C30 scores representative of the general population [23]. *Abbreviations*: *C5i*, C5-inhibitor; *ECU*, eculizumab; *EORTC QLQ-C30*, European Organization for Research and Treatment of Cancer Quality of Life Questionnaire Core 30; *FACIT*, Functional Assessment of Chronic Illness Therapy; *RAV*, ravulizumab

The mean FACIT-Fatigue scores recorded from the survey participants receiving ECU or RAV therapy were lower (ECU, 29.3 ± 14.0; RAV, 33.3 ± 13.0) compared to what has been reported for the general US population (43.6) (Fig. 2) [24]. Participants receiving ECU and RAV reported an average score of 62.4 (± 21.1) and 67.2 (± 19.0) for global health status on the EORTC QLQ-C30, respectively, compared to a general population score of 75.7 [23] (Fig. 2). The physical functioning scores were 76.4 (± 17.5) for ECU users and 76.7 (± 20.3) for RAV users, which were lower than the reported average of the general population at 91.0 (Fig. 2) [23]. Participants also reported scores lower than the population average for functioning related to role, emotional, cognitive, and social parameters of the EORTC QLQ-C30 (Online Resource 1 Figure).

### Healthcare resource utilization related to PNH

Among those survey respondents treated with ECU or RAV who had visited the ER (ECU, *n* = 19; RAV, *n* = 37) or had been hospitalized (ECU, *n* = 7; RAV, *n* = 25), an average of 1.05 (± 1.03) or 1.22 (± 1.08) PNH-related ER visits and 0.57 (± 0.53) or 1.36 (± 1.22) hospitalizations, respectively, were reported (Table 2). Common reasons (experienced by at least 25% of total responders) for PNH-related ER visits and hospitalizations included fatigue, breakthrough hemolysis, abdominal pain, and shortness of breath. Survey data also revealed that nearly all patients with PNH-related ER visits due to fatigue were anemic (Hb levels ≤ 12 g/dL, *n* = 16/17, 1 patient did not know their Hb level).

**Table 2:** PNH-related healthcare resource utilization (HCRU) within the past 12 months, among patients who had reported all-cause HCRU

**Number of PNH-related events**^**a**^	**Total** **Mean (SD)**	**Eculizumab** **Mean (SD)**	**Ravulizumab** **Mean (SD)**
PNH-related ER visits (*n* = 56; ECU = 19, RAV = 37)	1.16 (1.06)	1.05 (1.03)	1.22 (1.08)
PNH-related hospitalizations (*n* = 32; ECU = 7, RAV = 25)	1.19 (1.15)	0.57 (0.53)	1.36 (1.22)
**Reasons for PNH-related ER visits**^**b**^	**Total** (*n* = 39) **n (%)**	**Eculizumab** (*n* = 12) **n (%)**	**Ravulizumab** (*n* = 27) **n (%)**
Fatigue	17 (43.6%)	5 (41.7%)	12 (44.4%)
Breakthrough hemolysis	14 (35.9%)	3 (25.0%)	11 (40.7%)
Shortness of breath	12 (30.8%)	5 (41.7%)	7 (25.9%)
Abdominal pain	13 (33.3%)	6 (50.0%)	7 (25.9%)
**Reasons for PNH-related hospitalization**^**b**^	**Total** (*n* = 24) **n (%)**	**Eculizumab** (*n* = 4) **n (%)**	**Ravulizumab** (*n* = 20) **n (%)**
Fatigue	7 (29.2%)	1 (25.0%)	6 (30.0%)
Breakthrough hemolysis	9 (37.5%)	1 (25.0%)	8 (40.0%)
Shortness of breath	7 (29.2%)	3 (75.0%)	4 (20.0%)
Abdominal pain	8 (33.3%)	1 (25.0%)	7 (35.0%)

^a^All 122 patients were surveyed for HCRU. Those who reported an all-cause ER visit (*n* = 56) or all-cause hospitalization (*n* = 32) were queried for PNH-related HCRU

^b^Reasons for PNH-related ER visit or hospitalization that were reported by ≥ 25% of affected survey participants

*Abbreviations*: *ER*, emergency room; *PNH*, paroxysmal nocturnal hemoglobinuria; *SD*, standard deviation

### Analysis of work productivity and activity impairment

Out of the 122 individuals surveyed, 53 (43.4%) reported being in gainful employment (Table 3). Compared to RAV users, patients receiving ECU therapy displayed significantly higher (*p* < 0.05) values for absenteeism (ECU, 19.0%; RAV, 6.9%), presenteeism (ECU, 42.2%; RAV, 25.9%), and work productivity impairment (ECU, 48.4%; RAV, 30.2%). The daily activity impairment among survey respondents appeared to be similar between ECU and RAV groups at 43.1% and 37.7%, respectively.

**Table 3:** Employment status, work productivity, and activity impairment scores, and frequency of individuals with work impairment

**Employment status**	**Total** **n (%)**	**Eculizumab** **n (%)**	**Ravulizumab** **n (%)**
Employed (*N* = 122; ECU = 35, RAV = 87)	53 (43.4%)	18 (51.4%)	35 (40.2%)
**WPAI category**	**Mean % (SD)**	**Mean % (SD)**	**Mean % (SD)**
Absenteeism^a,b^* (*n* = 52; ECU = 18, RAV = 34)	11.1% (17)	19.0% (24)	6.9% (11)
Presenteeism^a,c^* (*n* = 52; ECU = 18, RAV = 34)	31.5% (27)	42.2% (32)	25.9% (23)
Work productivity impairment^a^* (*n* = 52; ECU = 18, RAV = 34)	36.5% (29)	48.4% (34)	30.2% (25)
Daily activity impairment (*N* = 122; ECU = 35, RAV = 87)	39.3% (27)	43.1% (27)	37.7% (26)
**Frequency of PNH patients with work or activity impairment**	**n (%)**	**n (%)**	**n (%)**
Employed individuals reporting hours missed from work in the past 7 days (*n* = 53; ECU = 18, RAV = 35)	25 (47.2%)	11 (61.1%)	14 (40.0%)
Employed individuals reporting affected productivity while at work^a^ (*n* = 52; ECU = 18, RAV = 34)	42 (80.8%)	15 (83.3%)	27 (79.4%)
Patients reporting activity impairment (*N* = 122; ECU = 35, RAV = 87)	107 (87.7%)	31 (88.6%)	76 (87.4%)

^a^Responses from one employed participant were not recorded because this participant had missed work for reasons unrelated to a health problem

^b^Absenteeism: Percentage of work time missed in the past 7 days because of one’s health problem [29]

^c^Presenteeism: Percentage of impairment experienced at work (reduced on-the-job effectiveness) in the past 7 days because of one’s health problem [29]

^*^Indicates significant difference between eculizumab and ravulizumab (*p* < 0.05)

*Abbreviations*: *PNH*, paroxysmal nocturnal hemoglobinuria; *SD*, standard deviation; *WPAI*, work productivity and activity impairment

Among the 53 individuals who were employed, 47.2% (*n* = 25/53) reported missing hours from work in the past 7 days. A large portion (80.8%, *n* = 42/52) of patients with PNH receiving therapy with ECU or RAV experienced an effect on their productivity at work due to their health problems. A majority of survey participants (87.7%, *n* = 107/122) also reported impairment in their daily activities related to their health problems (Table 3).

## Discussion

The current study reports the results of a survey that was applied to evaluate the burden of illness and QoL among 122 individuals that were living with a self-reported diagnosis PNH and receiving treatment with C5i therapies at the time of survey completion. Our findings demonstrate that many patients with PNH treated with ECU or RAV experience a substantial burden of illness, as measured by self-reported clinical parameters, symptoms of PNH, and psychometrically validated scales documenting a reduced QoL, despite treatment. While other studies have reported a decrease in QoL for individuals in the PNH population as a whole [8, 32], this study emphasizes that the current treatment options for PNH do not completely ameliorate the impact that the disease has on patient QoL, thus underscoring the need for improved therapies for the treatment of PNH. Furthermore, this study contributes to the limited body of information available on the real-world outcomes of PNH treatment with C5i. This is particularly true for the more recently available treatment approach with RAV, which has been the subject of fewer outcomes-related studies compared to ECU.

The results from our study describe important findings related to the patient experience for individuals living with PNH. For example, information regarding increased dosing with C5i therapy remains limited in the literature. Results from studies of ECU treatment in patients with PNH have suggested that very few patients require doses that are higher than the label recommendation [33, 34]. However, a recent study of ECU dosing in 707 patients with PNH using provider-based claims data found that 45.9% of ECU-treated PNH patients were on a dose higher than the label recommendation [35]. Analysis of our survey data on dosage characteristics demonstrated similar results and showed that nearly one-third of patients receiving ECU therapy were being treated with a dose that was higher than that recommended by the US label. The results from our study also provide insight on RAV dosing in patients with PNH, as similar findings were observed in RAV-treated patients with 33.3% of individuals in the 40–60 kg weight range and 18.2% of the individuals in the 60–100 kg weight range reporting treatment with doses of RAV that were higher than the US label recommendation. These findings suggest that there may be more patients with PNH relying on higher than label recommended doses of C5i therapy than previously appreciated. Furthermore, this observation, combined with the finding that many of these patients with PNH are not symptom-free, suggests that treatment of PNH with C5i is suboptimal.

It is important to note that the dosing intervals captured by the survey for ECU-treated patients may reflect issues with treatment compliance among this group. Current dosing recommendations for ECU advise that following the initial phase of treatment, patients with PNH should receive an IV infusion of ECU every 14 days [30]. The dosing intervals captured by our survey demonstrate that the average time between infusions for participating ECU-treated patients was 17.1 (± 14.7) days. Therefore, the extended period between ECU infusions reported by the study participants and the wide distribution of values provided may indicate poor treatment compliance among this population.

Responses collected about the clinical manifestations of PNH demonstrated that individuals receiving C5i therapy still experienced thrombotic events and had to rely on RBC transfusions despite receiving one or more years of treatment with ECU or RAV. In fact, among those patients on treatment for one or more years and who had been transfused at least once in their life, one in two ECU patients and one in four RAV patients required ≥ 1 RBC transfusions within the last 12 months. These findings may indicate that clinical interventions are required due to low Hb levels reported by survey participants. Hb levels ≤ 13 g/dL or ≤ 12 g/dL are considered anemic for men and non-pregnant women, respectively [36, 37]. The survey found that 87.5% of ECU users and 82.9% of RAV users reported Hb levels ≤ 12 g/dL and were therefore anemic, despite C5i therapy. In addition to collecting information on anemia and transfusion dependence, this study also evaluated thrombotic events among survey participants that had experienced at least one thrombotic event in their lifetime and had received C5i therapy for one or more years. This analysis demonstrated that 10.0% of ECU patients and 23.5% of RAV patients had experienced a thrombotic event within the past 12 months. These findings related to transfusions, anemia, and thrombotic events suggest that disease activity is still present in patients with PNH receiving treatment with ECU or RAV. This ongoing disease activity may suggest underlying EVH in patients with PNH treated with C5i, which is consistent with previous studies [13] and should be investigated further in PNH patients.

Findings related to patient-reported thrombotic events, transfusion history, and Hb levels are further corroborated by the PNH symptoms reported by survey participants. A large portion of respondents (ECU, 88.6%; RAV, 74.7%) reported they were currently experiencing fatigue symptoms, with significantly greater fatigue in patients with Hb levels < 10.5 g/dL versus those with Hb ≥ 10.5 g/dL (86.6% versus 52.2%). While treatment guidelines indicate that patients with PNH may experience a disconnect between fatigue levels and Hb concentration [38], two recent studies found a high correlation between fatigue and Hb levels in patients with PNH [39, 40], similar to our finding. Greater than one-third of the cohort also reported other current symptoms of PNH including breakthrough hemolysis, shortness of breath, headaches, difficulty focusing, sleeping difficulties, and back pain. A 2020 study that was conducted using data collected from the International PNH Registry identified similar proportions of patients with PNH reporting symptoms of fatigue (80.9%) and shortness of breath (45.3%); however, the data analysis was not stratified by current therapy [32]. Therefore, the results from our study provide new insight into the patient experience of individuals living with PNH receiving C5i therapy and suggest that many patients with PNH continue to experience PNH-related symptoms despite treatment with ECU or RAV.

The survey results also present information regarding C5i-treated patients and their QoL. Lower values on the FACIT-Fatigue scale indicate more severe fatigue, and a meaningful difference in fatigue is defined by a 3-point change in scores with this assessment [41]. When evaluating FACIT-Fatigue scores based on population norms, survey participants receiving C5i therapy reported FACIT-Fatigue scores (ECU, 29.3; RAV, 33.3) considerably lower than the mean score for the general US population (43.6) [24], indicating severe symptoms of fatigue among ECU and RAV users. The FACIT-Fatigue scores measured in survey respondents receiving ECU or RAV therapy are similar to what has been reported for the PNH population previously [8]; however, those analyses did not distinguish FACIT-Fatigue scores among patients with PNH by therapy. Our study also assessed QoL using the psychometrically tested and validated EORTC QLQ-C30 instrument. Higher functional and global health scale values measured by the EORTC QLQ-C30 instrument indicate better functioning, and clinically meaningful differences are represented by a 10-point difference in scores [42–45]. Survey participants with PNH reported mean EORTC QLQ-C30 scores > 10 points below the general population mean for global health status, physical, role, emotional, cognitive, and social functioning [23], indicating impaired QoL. The EORTC QLQ-C30 scores recorded by our survey are comparable to what has been previously reported for the PNH population as a whole [8].

In addition to the reduced QoL scores reported by the survey respondents, the effect of PNH symptoms on the survey participants’ well-being may also be reflected by their HCRU. Among those survey respondents who had reported all-cause HCRU, an average of one ER visit and one hospitalization was related to PNH in the past 12 months. These results suggest continued dependence on healthcare resources even though these patients report consistent treatment with ECU or RAV.

Results from the WPAI questionnaire indicated that survey participants on C5i therapy also experienced time missed at work and impairments in work productivity and daily activities. Greater than 80% of the employed survey participants reported reduced work productivity and overall activity impairment. Nearly half (47.2%) of respondents also reported missing hours from work in the past 7 days prior to completion of the survey. Therefore, patients with PNH, despite ECU or RAV treatment, continue to experience symptoms which impair their work productivity and daily activity.

The results from this survey demonstrate that the symptoms and clinical manifestations of PNH remain present in individuals receiving treatment with ECU or RAV. This study also indicates that the symptoms of PNH experienced by these patients are significant enough to have an impact on QoL and work productivity. The observed burden of illness among patients with PNH receiving ECU or RAV therapy may be a result of the effect that C5i has on the two distinct pathways of hemolysis that occur during PNH. Both ECU and RAV inhibit C5 activity, preventing the formation of MAC and subsequent IVH. However, EVH mediated by C3 also occurs during PNH, and it has been demonstrated that EVH is not prevented by C5i therapy [13]. These results suggest that C5i therapy and the prevention of IVH alone are not sufficient to completely ameliorate the symptoms and burden of illness experienced by patients with PNH.

Therapies that target more proximal mediators in the complement pathway, thus preventing both EVH and IVH, may represent more effective treatment for PNH. These proximal complement mediators that may also be targets for effective PNH therapy include C3, Factor B, and Factor D [46]. A C3-inhibiting therapy (pegcetacoplan — Apellis Pharmaceuticals) that prevents both IVH and EVH was recently approved by the Food and Drug Administration [47]. A recent phase 3, randomized, controlled trial known as the PEGASUS study (NCT03500549) enrolled patients with PNH who remained anemic (Hb ≤ 10.5 g/dL) despite stable ECU treatment (≥ 3 months) and compared pegcetacoplan treatment to ECU treatment in this population. Pegcetacoplan demonstrated superiority to ECU in the primary endpoint which measured change from baseline in Hb levels [48]. Improved QoL and fatigue were also measured among pegcetacoplan users as determined by the EORTC QLQ-C30 and FACIT-Fatigue questionnaires [49]. Results of the PEGASUS study suggest that the use of inhibitors against proximal mediators within the complement pathway may contribute to improved outcomes in patients with PNH by preventing both EVH and IVH.

There are some limitations to this study that must be considered. The sample size obtained for this survey (122 individuals) is a fairly small population. However, it is important to note that PNH is a rare disease, which creates challenges for recruiting a large population of survey participants. Similar to other surveys, missing responses could impact the results; however, prompts were built into the electronically administered survey to avoid missing data. Additionally, the survey is subject to selection bias where patients dissatisfied with their current C5i therapy may be more motivated to participate. This study was also limited by the subjectivity of patient-reported outcomes. As the survey relied on self-reporting, errors with recall, interpretation of what defines a symptom, or other response biases may have introduced measurement error. However, while claims analyses are often considered to be more objective compared to surveys using self-reported diagnosis, studies have identified a discordance in claims, as algorithms and claims reporting also have considerable variability in estimating diagnosis and healthcare encounters [50]. Claims analyses are also unable to capture specific data related to the patient perspective and QoL. Participation in the survey was voluntary; therefore, demographics (i.e., age and gender) were not controlled for in the study. Additionally, variables in the survey (i.e., PNH diagnosis, age, and gender) were not confirmed by a physician. The results from this study may not be generalizable to the total population of patients with PNH because of the convenience sampling used to obtain patient responses. Furthermore, recruitment through the PNH patient advocacy group could have led to sampling bias, although this foundation does represent a large portion of patients in the US. However, the present study population was similar in terms of patient characteristics and age at diagnosis when compared to a recent report that characterized the general PNH population [32]. Despite these limitations, this study represents one of the largest survey-based studies of patients with PNH in the US to date and contributes to the need for a larger collection of data related to patients with PNH, their QoL, and the response to current therapies available for the treatment of this disease.

## Conclusions

Results from this survey demonstrate that many patients with PNH reciveing treatment with ECU and RAV continue to experience disease symptoms such as fatigue, display clinical manifestations such as anemia, and thrombotic events, and continue to rely on RBC transfusions. The patient responses collected by the survey also indicate a significant burden in terms of QoL, work productivity, and HCRU for individuals living with PNH and treated with C5i therapies. Overall, our results suggest that patients with PNH receiving therapy with ECU or RAV maintain a substantial burden of illness, indicating that there is a need for novel therapeutics in PNH treatment.

## Supplementary Information

Below is the link to the electronic supplementary material.

Supplementary file1 (PDF 251 KB)

## Data Availability

All relevant summary data are provided in the manuscript text, tables, and figures.
