# Supplementary material for: The burden of illness in patients with paroxysmal nocturnal hemoglobinuria receiving treatment with the C5-inhibitors eculizumab or ravulizumab: results from a US patient survey
Source: Ann Hematol. 2022 Jan 1;101(2):251–63. doi: 10.1007/s00277-021-04715-5 (PMC8720163; doi:10.1007/s00277-021-04715-5)
Supplement: Supplementary file 1 — Supplementary file1 (PDF 251 KB) [file 277_2021_4715_MOESM1_ESM.pdf]

Online Resource 1

Annals of Hematology

The Burden of Illness in Patients with Paroxysmal Nocturnal Hemoglobinuria Receiving Treatment with the C5-Inhibitors Eculizumab or Ravulizumab: Results from a United States Patient Survey

David Dingli, MD, PhD[1], Joana E. Matos, PhD, Kerri Lehrhaupt, BSc, Sangeeta Krishnan, PharmD, MS, Michael Yeh, MD, MBA, MPH, Jesse Fishman, PharmD, MSc, Sujata P. Sarda, PhD, Scott B. Baver, PhD

Corresponding Author: David Dingli, MD, PhD

Corresponding Author Affiliation: [1]Mayo Clinic, Rochester, MN, USA

Email: dingli.david@mayo.edu

Contents:
This supplementary file includes the Online Resource 1 Figure, which presents the EORTC QLQ-C30 functioning scores recorded by the survey.

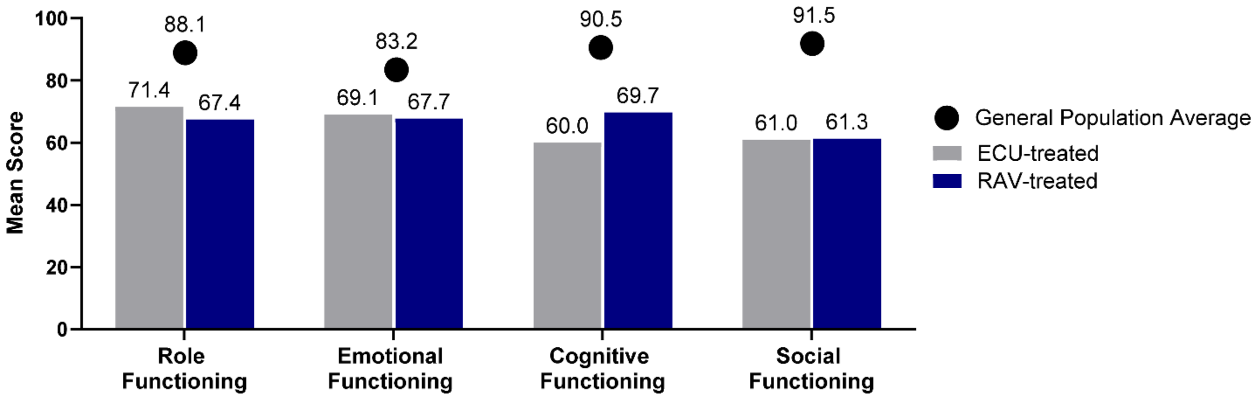

Online Resource 1 Figure: EORTC QLQ-C30 Scores for ECU and RAV users

Mean EORTC-QLQ-C30 scores for role, emotional, cognitive, and social functioning recorded from the patients with PNH (N=122) and compared to EORTC QLQ-C30 scores obtained from the general population [23]

Abbreviations: ECU, eculizumab; EORTC QLQ-C30, European Organization for Research and Treatment of Cancer Quality of Life Questionnaire Core 30; *PNH, paroxysmal nocturnal hemoglobinuria; RAV, ravulizumab*

References:

23. Hinz A, Singer S, Brahler E (2014) European reference values for the quality of life questionnaire EORTC QLQ-C30: Results of a German investigation and a summarizing analysis of six European general population normative studies. Acta Oncol 53 (7):958-965. doi:10.3109/0284186X.2013.879998
